# Genetic diversity and *Wolbachia* infection in the Japanese encephalitis virus vector *Culex tritaeniorhynchus* in the Republic of Korea

**DOI:** 10.1186/s13071-024-06595-w

**Published:** 2024-12-18

**Authors:** Jiseung Jeon, Heung Chul Kim, Martin J. Donnelly, Kwang Shik Choi

**Affiliations:** 1 Department of Biology, College of Natural Sciences, Kyungpook National University, Daegu, 41566 Republic of Korea; 2 BK21 FOUR KNU Creative BioResearch Group, School of Life Sciences, Kyungpook National University, Daegu, 41566 Republic of Korea; 3 U Inc., Gasan Digital 2-ro, Geumcheon-gu, Seoul, 08504 Republic of Korea; 4 Department of Vector Biology, Liverpool School of Tropical Medicine, Liverpool, L3 5QA UK

**Keywords:** *Culex tritaeniorhynchus*, Japanese encephalitis virus, Genetic diversity, *Wolbachia*, Republic of Korea

## Abstract

**Background:**

*Culex tritaeniorhynchus*, a major vector of Japanese encephalitis virus (JEV), is found across a broad geographical range, including Africa, Asia, Australia and Europe. Understanding the population structure and genetic diversity of pathogen vectors is increasingly seen as important for effective disease control. In China and Japan, two countries in close proximity to the Republic of Korea (ROK), *Cx. tritaeniorhynchus* has been categorized into two clades based on the DNA barcoding region of mitochondrial cytochrome *c* oxidase subunit I (*COI*), suggesting the presence of cryptic species. No comprehensive analysis of the genetic diversity in *Cx. tritaeniorhynchus* has been conducted in the ROK. To address this gap, we investigated the population structure of *Cx. tritaeniorhynchus* in the ROK.

**Methods:**

In Daegu, mosquito collections were conducted over a 2-year period from 2022 to 2023. For all other regions, *Cx. tritaeniorhynchus* specimens collected in 2023 were used. The *COI* barcoding region was analyzed to determine the genetic structure of the populations, supplemented with data from the 28S ribosomal DNA region. Each population was also examined for the eventual presence of *Wolbachia* infection. Finally, a back trajectory analysis was conducted to assess the possibility of international introduction of *Cx. tritaeniorhynchus* into the ROK.

**Results:**

The analysis of the *COI* region revealed the presence of two distinct clades within *Cx. tritaeniorhynchus*; these clades were the same as *Cx. tritaeniorhynchus *continental type (Ct-C) and *C. tritaeniorhynchus* Japanese type (Ct-J) previously reported. In contrast, the nuclear 28S region showed no significant genetic differentiation between these clades. *Wolbachia* infection was confirmed in some populations, but there was no evidence of an association with *Wolbachia* in Ct-C and Ct-J. It was also confirmed that the ROK is currently dominated by the Ct-J clade, with a possible introduction of Ct-C via air currents.

**Conclusions:**

Determining the presence of cryptic species is important for preventing vector-borne diseases. The results of this study confirm the existence of two clades of *Cx*. *tritaeniorhynchus* in the ROK, with Ct-J being the dominant clade. Our findings enhance current understanding of the genetic diversity within *Cx. tritaeniorhynchus* and provide valuable insights for the prevention of JEV outbreaks and the effective management of *Cx. tritaeniorhynchus* populations in East Asia.

**Graphical Abstract:**

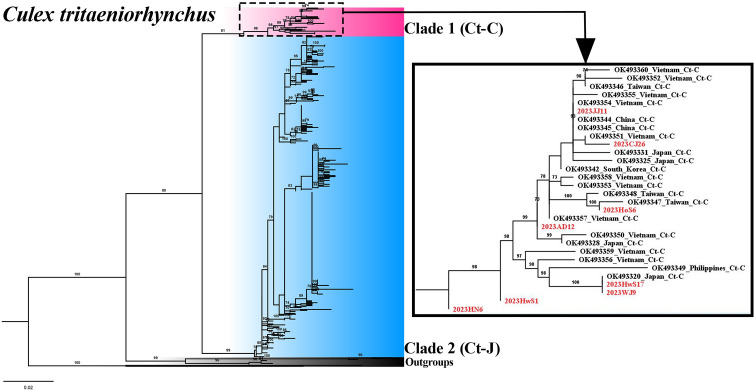

**Supplementary Information:**

The online version contains supplementary material available at 10.1186/s13071-024-06595-w.

## Background

*Culex tritaeniorhynchus* Giles, 1901 is a mosquito species with a global distribution that includes Africa, Asia, Australia and Europe [1, 2] and is a primary vector of Japanese encephalitis virus (JEV). Enzootic transmission of JEV occurs through amplifying hosts, such as pigs and water birds. In most cases, the symptoms of Japanese encephalitis in individuals infected with JEV are mild, but severe clinical illness occurs in some patients [3]. It has been estimated that 100,000 cases of Japanese encephalitis occur annually at the global level, resulting in approximately 25,000 deaths [3]. In the Republic of Korea (ROK), thousands of cases of JEV were recorded annually between the first reported outbreak of JEV in the 1940s up to the 1960s. The number of cases has decreased significantly to approximately 20 to 40 cases per year with the introduction of a human vaccine in the 1970s [4]. However, the emergence of JEV genotypes not previously present in the ROK underscores the need for ongoing surveillance and research on *Cx. tritaeniorhynchus* [5–7].

The *Cx. vishnui* subgroup, which includes *Cx. tritaeniorhynchus*, *Cx. vishnui* Theobald, 1901 and *Cx. pseudovishnui* Colless, 1957, are all capable of transmitting JEV [8–11]. *Culex tritaeniorhynchus* is the dominant species in the ROK and is currently recognized as the primary vector of JEV [12]. A recent study conducted in China and Japan revealed that *Cx. tritaeniorhynchus* is divided into two clades based on mitochondrial DNA (mtDNA) analysis [13, 14]. Arai et al. [14] suggested that the clades were possibly indicators of cryptic species and proposed that *Cx. tritaeniorhynchus* is divided into *Cx. tritaeniorhynchus* Japanese type (Ct-J), predominantly found in Japan, and *C. tritaeniorhynchus *continental type (Ct-C), distributed on continents other than Japan. These authors further suggested that Ct-C may have been introduced into Japan through migration on long-distance air currents [14].

The first aim of this study was to determine the distribution of mtDNA clades and determine if there was evidence for any additional population structuring. Cryptic species are frequently observed in mosquito genera, including *Anopheles*, *Aedes* and *Culex* [15, 16], and accurate species identification is crucial for developing effective, targeted vector control strategies [16]. Arai et al. [14] suggested that both Ct-J and Ct-C types may coexist in the ROK, unlike in Japan. However, their study was based on a limited number of *Cx. tritaeniorhynchus* samples collected in the ROK. Therefore, comprehensive sampling and analysis across various regions of the ROK are essential to validate previous findings and to inform efficient vector management strategies.

The cytochrome* c* oxidase subunit I (*COI*) region is a commonly used genetic marker for accurate species identification, serving as a barcoding region [17], and has been used to identify mosquito cryptic species [18–20]. Koh et al. [21] recently conducted a comparative analysis of ribosomal DNA (rDNA) and *COI* regions in 33 mosquito species. Their study demonstrated that rDNA regions (18S, 28S) are also valuable tools for phylogenetic analysis and can help identify intraspecific variation that may be overlooked using *COI* regions alone. Additionally, rDNA regions can prove useful for studying geographically isolated conspecifics [21].

We also sought to determine whether *Wolbachia* infection segregated with mtDNA clade. *Wolbachia* is a cytoplasmically inherited bacterium that has been identified in numerous insect species, including mosquitoes [22, 23]. *Wolbachia* infection is often associated with cytoplasmic incompatibility of eggs and sperm, which can create a reproductive barrier [24, 25]. While the role of *Wolbachia* in speciation and the formation of cryptic species remains controversial [16], recent studies suggest a potential link between *Wolbachia* and cryptic species in some mosquitoes of the genera *Aedes* and *Culex* [26–28]. A study conducted in China reported that 17.1% of *Cx. tritaeniorhynchus* mosquitoes were infected with *Wolbachia* supergroup B [29] but the authors did not determine whether the *Wolbachia* infection was associated with the two clades (Ct-C and Ct-J) of *Cx. tritaeniorhynchus*.

The final aim of the study was to investigate whether there was evidence for introductions of mtDNA clades into the ROK. The brown planthopper (*Nilaparvata lugens*), which causes significant damage to agricultural crops in the ROK, is believed to travel to the ROK from Southeast Asia and China via air currents [30]. This hypothesis has been validated through back trajectory analysis, which tracks the origin of airflow [31]. One of the advantages of back trajectory analysis is the capability to track a specific particle's path backwards. Since invasive pests often have unknown origins, back trajectory analysis can help identify where the pest might have originated [14, 31]. The spread of vector-borne diseases through air currents can also be seen in the case of *Anopheles* mosquitoes [32]. No studies have specifically investigated the potential introduction of *Cx. tritaeniorhynchus* into the ROK through air currents from other countries. However, given the case of the brown planthopper and the remarkable flight capabilities of *Cx. tritaeniorhynchus* [14], it is plausible that this mosquito species could also be introduced via long-distance flights.

## Methods

### Mosquito collection and identification

**Fig. 1:**
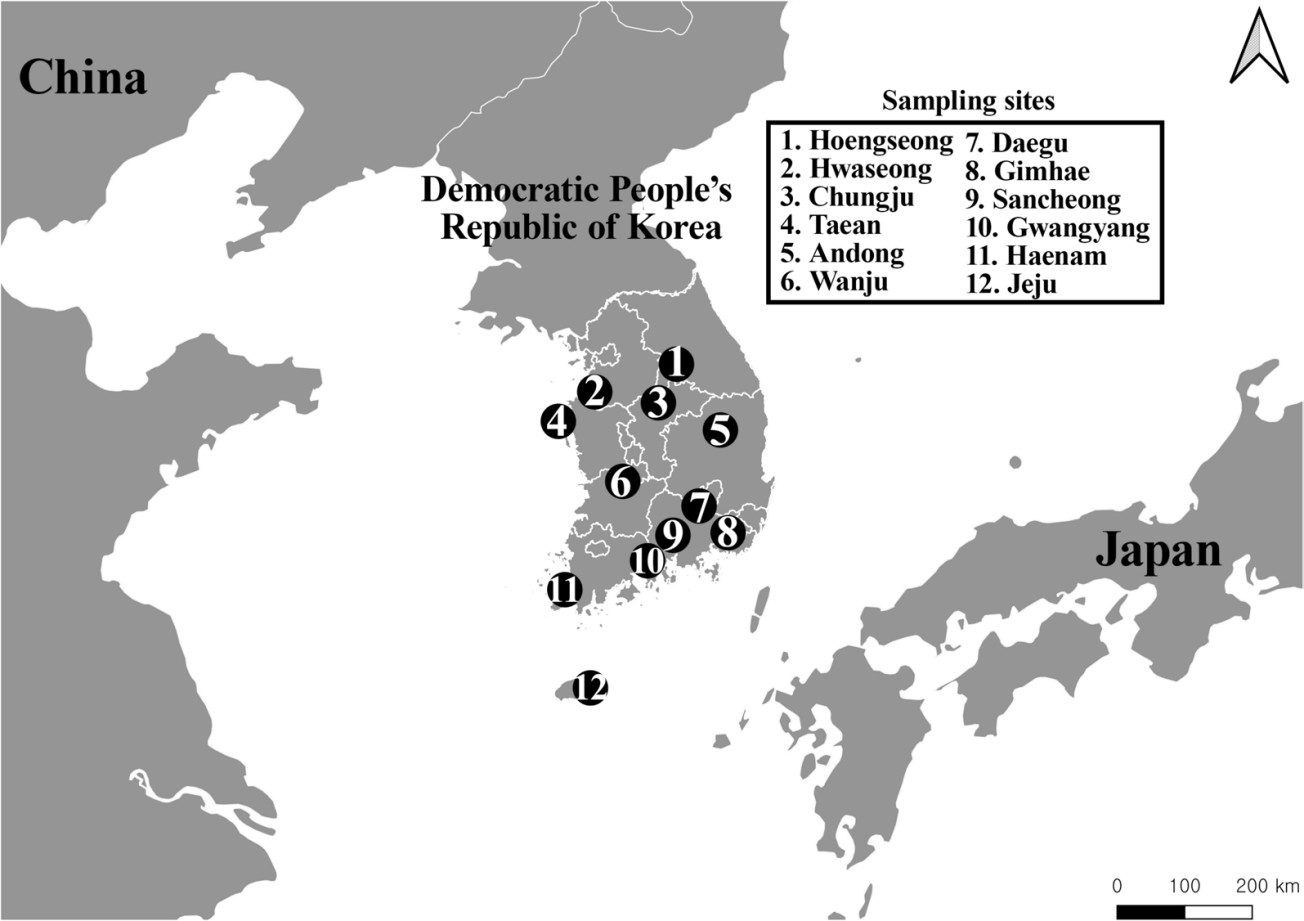
Collection sites of *Culex tritaeniorynchus* within the Republic of Korea used in this study. This map was generated using QGIS v. 3.26.3 (https://www.qgis.org/ko/site). Details of the sampling sites are presented in Additional file 1: Table S1

Collections were conducted from July to September at 12 locations in the ROK, specifically from areas near cowsheds based on the known habitat preference of the species (Fig. 1; Additional file 1: Table S1). Collections in Daegu were conducted over a 2-year period (2022–2023) at the same site, but in 2023 specimens from all the other regions were collected (Fig. 1). Mosquitoes were collected using a black light trap (BT Global, Seongnam, ROK) with dry ice as an additional attractant. The collected mosquitoes were transported to Kyungpook National University (Daegu, ROK) and stored at − 70 °C until identification. Specimens of *Cx. tritaeniorhynchus* were identified using a morphological identification key [33] and then stored at − 70 °C until DNA extraction. Subsequent experiments were conducted using only female mosquitoes.

### Mitochondrial DNA sequencing

DNA was extracted using the Clear-S™ Quick DNA Extraction Kit (InVirusTech, Gwangju, ROK) following the manufacturer’s protocol. The extracted genomic DNA was used to obtain data for the *COI* region. PCR amplification of the *COI* region was conducted using the universal primer pairs LCO1490 (5′-GGT CAA ATC ATA AAG ATA TTG G-3′) and HCO2198 (5′-TAA ACT TCA GGG TGA CCA AAA AAT CA-3′) [34]. The PCR reaction mixture (total volume 25.0 μl) included 1× PCR buffer, 0.2 mM dNTPs, 0.4 μM of each primer, 0.5 units of *Taq* DNA polymerase (TaKaRa, Shiga, Japan) and 1 μl of extracted genomic DNA. The PCR cycling conditions consisted of an initial denaturation at 94 °C for 5 min, followed by 35 cycles at 94 °C for 30 s, 58 °C for 30 s and 72 °C for 1 min, with a final extension at 72 °C for 5 min.

To confirm amplification, amplicons were assessed through electrophoresis in a 1.5% agarose gel. Samples that were successfully amplified underwent paired Sanger dideoxy sequencing using the universal primer pairs LCO1490 and HCO2198 (Macrogen, Daejeon, ROK). The *COI* sequences were aligned and trimmed using BioEdit [35], compared and verified using NCBI’s Basic Local Alignment Search Tool (BLAST). The *COI* sequences of all individuals of *Cx. tritaeniorhynchus* obtained in this study have been deposited in the NCBI GenBank (GenBank accession numbers: PP972404–PP972716).

### Ribosomal DNA sequencing

To obtain the rDNA sequence of *Cx. tritaeniorhynchus*, the 28S region was sequenced. The 28S rDNA sequences of *Cx. tritaeniorhynchus* deposited in NCBI GenBank were used for primer design (GenBank accession numbers: AF165896, AF165897, OM542404, OM542448). Primers were designed using Primer3 [36]. Two pairs of primers were newly designed for this study: (i) tri_28S_F1 (5′-AGG CCT CAA ATA ATG TGT GAC T-3′) and tri_28S_R1 (5′-CCG TAA TCC CGC ACA GTT TC-3′); and (ii) tri_28S_F2 (5′-GGG ACC CGT CTT GAA ACA C-3′) and tri_28S_R2 (5′-CTT CAA CGC ACA CCA CCA G-3′). The PCR reaction mixture (total volume 25.0 μl) included 1× PCR buffer, 0.2 mM dNTPs, 0.4 μM of each primer, 0.5 units of *Taq* HotStart DNA polymerase (TaKaRa) and 1 μl of extracted genomic DNA. The PCR cycling conditions consisted of an initial denaturation at 94 °C for 5 min, followed by 30 cycles at 94 °C for 30 s, 56–58 °C (56 °C: tri_28S_F1/tri_28S_R1; 58 °C: tri_28S_F2/tri_28S_R2) for 30 s and 72 °C for 1 min, with a final extension at 72 °C for 5 min.

Amplification was confirmed by electrophoresis in a 1.5% agarose gel. Samples that were successfully amplified underwent pair-end Sanger dideoxy sequencing using PCR primers (Macrogen). The 28S rDNA sequence of *Cx. tritaeniorhynchus*, which was validated using the same method as for the *COI* region, has been deposited in NCBI GenBank (GenBank accession numbers: PP974359–PP974374).

### Detection of *Wolbachia* infection

PCR methods were employed to detect *Wolbachia* infection in each population using genomic DNA extracted from *Cx. tritaeniorhynchus*. The detection of *Wolbachia* infection was performed in two steps. Initially, PCR was used to amplify the *Wolbachia* surface protein gene (*wsp*). For specimens that did not yield amplification of the *wsp* gene, additional PCR targeting the 16S rDNA region of *Wolbachia* was conducted. Samples that failed to amplify for 16S rDNA were considered to be negative for *Wolbachia* infection.

The PCR protocol followed for amplification of the *wsp* gene was that reported by Zhou et al. [23], using the primer pairs wsp_81F (5′-TGG TCC AAT AAG TGA TGA TGA AGA AAC-3′) and wsp_691R (5′-AAA AAT TAA ACG CTA CTC CA-3′). The PCR reaction mixture (total volume 25.0 μl) consisted of 1× PCR buffer, 0.2 mM dNTPs, 0.4 μM of each primer, 0.5 units of *Taq* HotStart DNA polymerase (TaKaRa) and 2 μl of extracted genomic DNA. The PCR cycling conditions consisted of an initial denaturation at 94 °C for 5 min, followed by 40 cycles at 94 °C for 30 s, 53 °C for 30 s and 72 °C for 1 min, with a final extension at 72 °C for 5 min.

PCR amplification of the 16S rDNA of *Wolbachia* was performed using primers reported by Werren and Windsor [37] (WF: 5′-CAT ACC TAT TCG AAG GGA TAG-3′; WR: 5′-AGC TTC GAG TGA AAC CAA TTC-3′). The reaction mixture for PCR amplification (total volume 25.0 μl) consisted of 1× PCR buffer, 0.2 mM dNTPs, 0.4 μM of each primer, 0.5 units of *Taq* HotStart DNA polymerase (TaKaRa) and 2 μl of extracted genomic DNA. The PCR cycling conditions consisted of an initial denaturation at 94 °C for 5 min, followed by 40 cycles at 94 °C for 30 s, 60 °C for 30 s and 72 °C for 1 min, with a final extension at 72 °C for 5 min.

Amplification of the *wsp* gene and 16S rDNA was confirmed by electrophoresis in 1.5% agarose gels. Samples that were successfully amplified were subjected to Sanger dideoxy sequencing (Macrogen). The sequences of all *wsp* genes detected in *Cx. tritaeniorhynchus* obtained in this study have been deposited in NCBI GenBank (GenBank accession numbers: *wsp*: PQ014158–PQ014189; 16S: PQ625809﻿–PQ625837; PQ579157–PQ579160).

### Back trajectory analysis

Back trajectory analysis was conducted to assess the potential introduction of *Cx. tritaeniorhynchus* individuals from overseas into the ROK. National Oceanic and Atmospheric Administration’s hybrid single-particle Lagrangian integrated trajectory (NOAA’s HYSPLIT) model was used for this analysis [38]. The analysis was performed using the web-based version (https://www.arl.noaa.gov/hysplit/), with data analyzed at 24-h intervals for 7 days starting from the date of *Cx. tritaeniorhynchus* (Ct-C) collection. Arai et al. [14] found that *Cx. tritaeniorhynchus* exhibits the longest flight time at 15 °C to 20 °C, with a decrease in flight time at 25 °C. In the ROK, the average temperature in August, the hottest month of the year, ranges from 19.7 °C to 26.7 °C. Therefore, the altitude of the arrival point was set to 500 m, considering that the temperature decreases by 0.65 °C per 100 m of elevation above the ground [39].

### Data analysis

Phylogenetic analysis was done using the *COI* sequences of *Cx. tritaeniorhynchus* individuals collected in this study, as well as on the *wsp* gene of *Wolbachia*. Sequences were aligned using the L-INS-i method in MAFFT v.7 [40]. Maximum likelihood (ML) analysis and substitution model selection were performed using the IQ-Tree web version (http://iqtree.cibiv.univie.ac.at/) [41–44]. Based on the Bayesian information criterion (BIC), the substitution model for each region was determined as follows: *COI*: TVM + F + I + G4; *wsp*: TVM + F + I. The bootstrap method with 1000 replicates was used to assess the support for the phylogenetic trees. For the *COI* region, sequences from Arai et al. [14] were included to differentiate between Ct-C and Ct-J types of *Cx. tritaeniorhynchus* in the ROK (Additional file 2: Table S2). The ML tree was visualized using Figtree v.1.4.4 (http://tree.bio.ed.ac.uk/software/figtree/).

The genetic diversity of the *COI* region was assessed, and the mismatch distribution (pairwise differences) for estimating changes in population size was analyzed using DnaSP v.6 [45]. Genetic diversity metrics, including the number of segregating sites (*S*), number of haplotypes (*H*), haplotype diversity (*H*_*d*_), average number of nucleotide differences (*k*) and nucleotide diversity (*π*), were calculated for each population of *Cx. tritaeniorhynchus*. Neutrality tests were performed using Arlequin v.3.5 [46], and Tajima’s *D* and Fu’s *F*_*s*_ values were calculated [47, 48]. To investigate genetic variation, an analysis of molecular variance (AMOVA) was conducted using Arlequin v.3.5 [46]. This analysis assessed variation among regions (northwest: Hwaseong + Taean; northeast: Chungju + Hoengseong; southwest: Haenam + Wanju + Gwangyang; southeast: Daegu + Gimhae + Sancheng + Andong; island: Jeju) and within populations. The northern region is defined by areas located near the western sea (Yellow Sea) in the northwest and those situated inland in the northeast. In the southern region, the Taebaek and Sobaek Mountains serve as significant geographical boundaries that delineate each area [49]. Based on this mountain range, the southeast and southwest regions were established. Additionally, a median-joining haplotype network analysis was performed to explore the relationship of each haplotype in the *COI* using PopART v.1.7 [50].

## Results

### Genetic diversity of *Cx. tritaeniorhynchus*

**Fig. 2:**
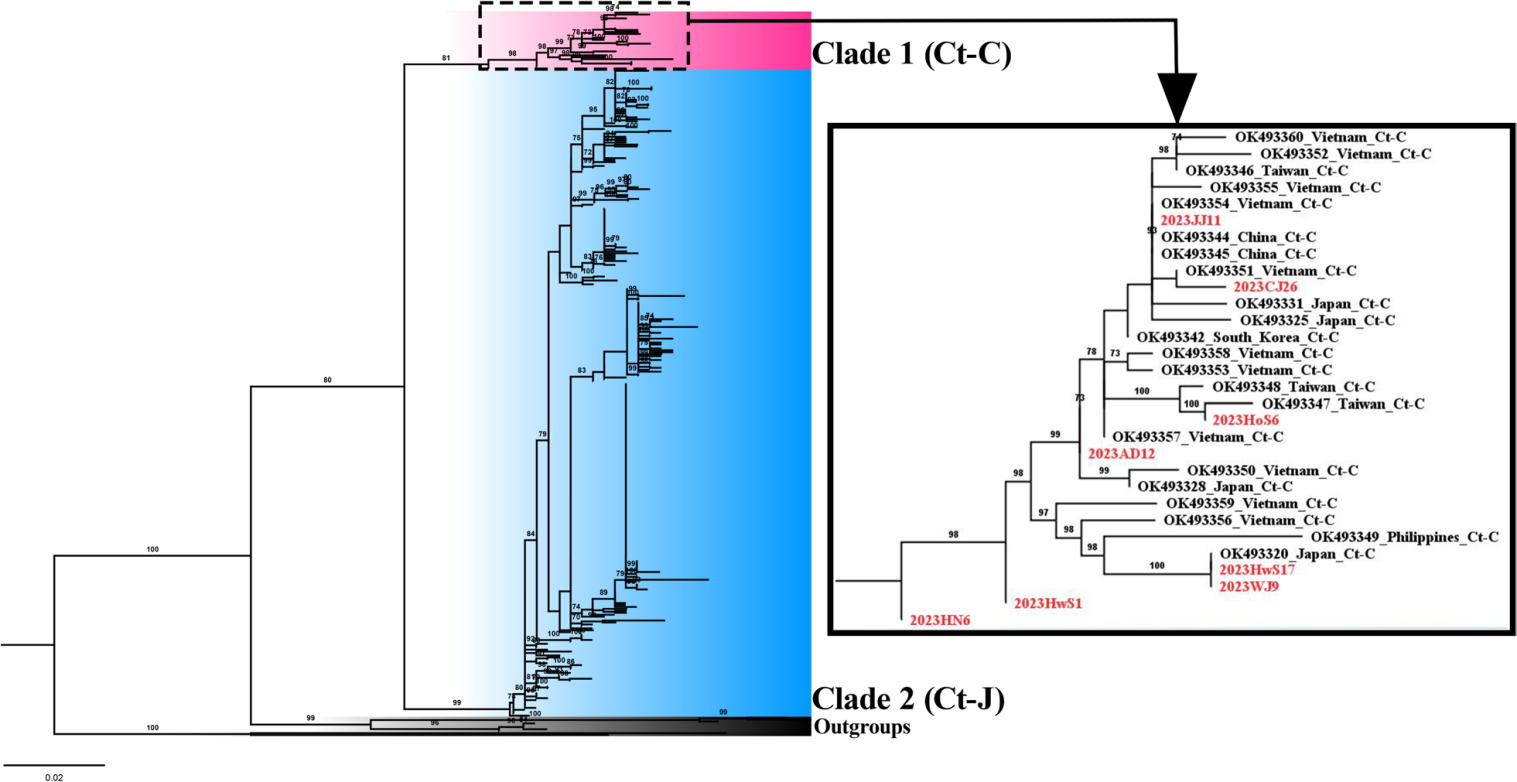
Maximum likelihood phylogenetic trees constructed in this study using the *COI* region of *Culex tritaeniorhynchus*. Bootstrap analysis was performed with 1000 replications, and the relative values are reported on the branch. Clade 1 (Ct-C) is highlighted in red, and clade 2 (Ct-J) is highlighted in blue. The subtree on the right represents individuals from clade 1 (Ct-C), with those collected in this study labeled in red (AD, Andong; CJ, Chungju; HN, Haenam; HoS, Hoengseong; HwS, Hwaseong; JJ, Jeju; WJ, Wanju). Information regarding the sequences used in this study is provided in Additional file 2: Table S2. Ct-C, *Cx. tritaeniorhynchus* continental type; Ct-J, *Cx. tritaeniorhynchus* Japanese type

**Fig. 3:**
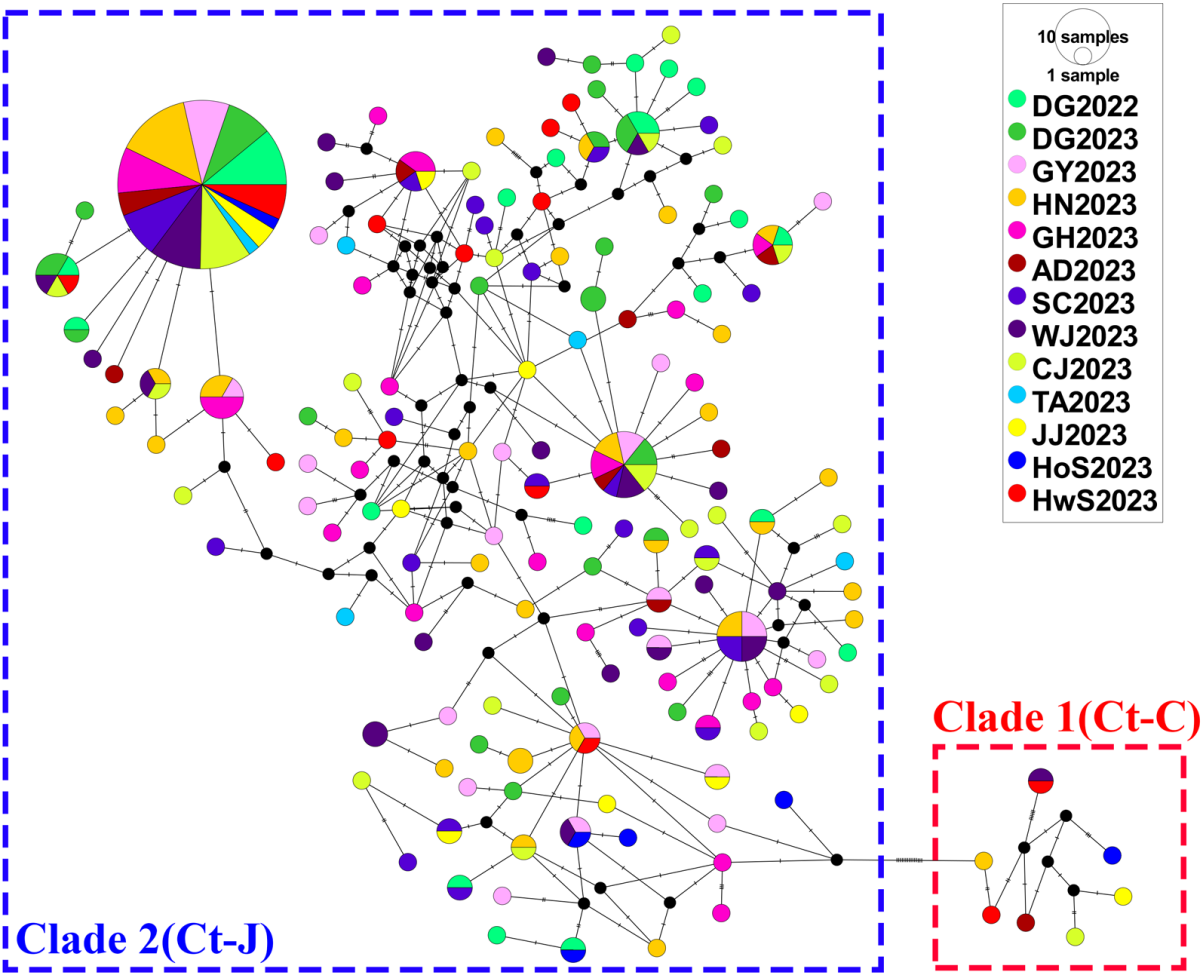
Median-joining haplotype network constructed using PopART v.1.7. Haplotypes are represented by circles, with the size of each circle being proportional to the sample size (AD, Andong; CJ, Chungju; HN, DG Daegu; GH, Gimhae; GY, Gwangyang; Haenam; HoS, Hoengseong; HwS, Hwaseong; JJ, Jeju; SC, Sancheong; TA, Taean; WJ, Wanju). Missing haplotypes are indicated by black circles. Lines connecting the circles are proportional to the number of mutation steps. Haplotypes belonging to clade 1 (Ct-C) are highlighted in red, and those belonging to clade 2 (Ct-J) are highlighted in blue. Ct-C, *Cx. tritaeniorhynchus* continental type; Ct-J, *Cx. tritaeniorhynchus* Japanese type

A total of 313 *COI* fragments (611 bp) were obtained from female *Cx. tritaeniorhynchus* collected from the 12 regions of the ROK. Phylogenetic analysis using ML methods confirmed the presence of both clades (Ct-C and Ct-J) in the ROK (Fig. 2) although of the 313 specimens analyzed, 305 belonged to Ct-J and only eight were classified as Ct-C. Only one female mosquito of Ct-C was identified in each of the Jeju, Chungju, Hoengseong, Andong, Wanju and Haenam sites, and two Ct-C individuals were identified among the 18 specimens collected in Hwaseong. No Ct-C individuals were found in the other collection regions despite extensive sampling. These findings indicate that at time of sampling the Ct-J clade was predominant in the ROK. Haplotype network analysis to infer the relationships among haplotypes within each clade revealed a complex haplotype network structure for *Cx. tritaeniorhynchus* (Fig. 3). Seven haplotypes were identified in the eight mosquitoes of the Ct-C clade. Similarly, 148 haplotypes were identified in the 305 mosquitoes of the Ct-J clade. The networks of Ct-C and Ct-J are separated by a significant number of mutations (*N* = 15), with most branches within Ct-J separated by only one or two mutation steps. Notably, a dominant haplotype, likely representing the ancestral form, was an important feature of Ct-J. This dominant haplotype was present in all regions sampled, with 91 of the 305 (29.8%) Ct-J individuals carrying this haplotype.

To measure the genetic diversity of Ct-C and Ct-J in each population, we identified a total of 155 haplotypes, with high haplotype diversity (*H*_*d*_ values ranging from 0.875 to 1.000) observed across all regions (Table 1). Among the Ct-J populations, the highest haplotype diversity was recorded in Gimhae (*H* = 21, *H*_*d*_ = 0.933), Taean (*H* = 5, *H*_*d*_ = 0.933) and Hoengseong (*H* = 5, *H*_*d*_ = 0.933), and the lowest was observed in Hwaseong (*H* = 11, *H*_*d*_ = 0.875). Tajima's *D* test did not yield statistically significant results for any of the populations (Table 1). However, Fu's *F*_*s*_ test revealed statistically significant negative values in five of the 12 regions (Chungju: *F*_*s*_ = − 5.99242, *P* < 0.05; Gimhae: *F*_*s*_ = − 6.05995, *P* < 0.05; Sancheong: *F*_*s*_ = − 6.94973, *P* < 0.05; Gwangyang: *F*_*s*_ = − 7.50043, *P* < 0.01; Haenam: *F*_*s*_ = − 10.86232, *P* < 0.01) (Table 1), suggesting an excessive number of rare alleles and a deviation from assumptions of neutrality.

**Table 1:** Genetic diversity of two clades of *Culex tritaeniorhynchus* found in the Republic of Korea

Sites	Clade^b^	Sample size (*n*)	Genetic diversity metrics^a^					Neutrality test statistics	
			*H*	*S*	*k*	*H*_*d*_	*π*	Tajima’s *D*	Fu’s *F*_*s*_
Hoengseong	1 (Ct-C)	1	–	–	–	–	–	–	–
	2 (Ct-J)	6	5	16	7.467	0.933	0.01222	0.40542	0.33219
Hwaseong	1 (Ct-C)	2	2	8	8.000	1.000	0.01309	0	2.07944
	2 (Ct-J)	16	11	24	7.742	0.875	0.01267	0.28696	− 1.32947
Chungju	1 (Ct-C)	1	–	–	–	–	–	–	–
	2 (Ct-J)	30	21	40	8.839	0.915	0.01447	− 0.45883	− 5.99242*
Taean	2 (Ct-J)	6	5	20	9.000	0.933	0.01473	0.17181	0.64306
Andong	1 (Ct-C)	1	–	–	–	–	–	–	–
	2 (Ct-J)	11	8	21	7.818	0.891	0.01280	0.41104	− 0.32738
Wanju	1 (Ct-C)	1	–	–	–	–	–	–	–
	2 (Ct-J)	30	19	34	8.391	0.910	0.01373	− 0.08134	− 4.11135
Daegu (2022)	2 (Ct-J)	29	19	36	8.958	0.887	0.01466	− 0.08385	− 4.01479
Daegu (2023)	2 (Ct-J)	31	20	36	8.501	0.931	0.01391	− 0.20603	− 4.74562
Gimhae	2 (Ct-J)	32	21	36	7.933	0.933	0.01298	− 0.40714	− 6.05995*
Sancheong	2 (Ct-J)	29	21	34	8.320	0.929	0.01362	− 0.14296	− 6.94973*
Gwangyang	2 (Ct-J)	30	21	35	7.384	0.931	0.01208	− 0.59986	− 7.50043*
Haenam	1 (Ct-C)	1	–	–	–	–	–	–	–
	2 (Ct-J)	44	28	38	7.878	0.913	0.01289	− 0.33891	− 10.86232*
Jeju	1 Ct-C)	1	–	–	–	–	–	–	–
	2 (Ct-J)	11	8	21	7.382	0.891	0.01208	0.13443	− 0.46671

*Statistically significant at *P* < 0.05)

^a^* H*, number of haplotypes; *S*, number of segregating sites; *k*, average number of nucleotide differences; *Hd*, haplotype diversity; *π*, nucleotide diversity

^b^1 (Ct-C), Clade 1 *Cx. tritaeniorhynchus* continental type; 2 (Ct-J), clade 2 *Cx. tritaeniorhynchus* Japanese type

**Fig. 4:**
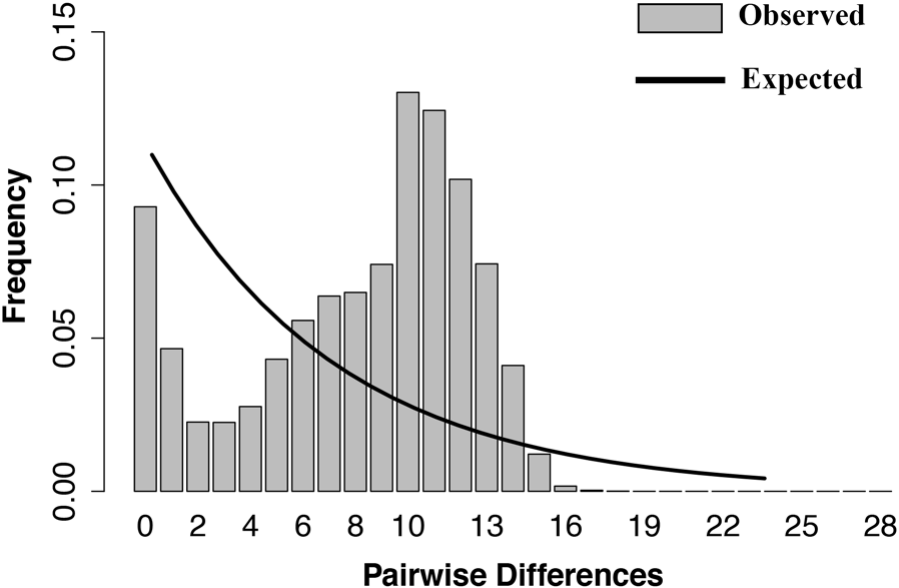
Mismatch distributions of *Culex tritaeniorhynchus* (Ct-J). Each gray bar represents the frequency of pairwise nucleotide differences between individuals. The black line indicates the expected value under a model of constant population size. Ct-J, *Cx. tritaeniorhynchus* Japanese type

In accordance with the results of Tajima’s *D* test, a mismatch analysis of the *COI* region revealed a ragged multimodal distribution (Fig. 4). A multimodal or bimodal distribution generally suggests a constant population size or demographic equilibrium. The AMOVA results indicated that most of the genetic variation in *Cx. tritaeniorhynchus* was attributable to within-population differences (100%), with minimal genetic variation observed among regions (Table 2).

**Table 2:** Analysis of molecular variance of *Culex tritaeniorhynchus* (Ct-J) in the ROK

Source of variation	*df*	Sum of squares	Variance components	Percentage of variation
Among regions	4	15.883	− 0.00282Va	− 0.07
Within populations	300	1234.772	4.11591Vb	100.07
Total	304	1250.655	4.11309	

Ct-J, *Cx. tritaeniorhynchus* Japanese type

To investigate the power of additional markers to resolve structure in *Cx. tritaeniorhynchus,* we sequenced the 28S rDNA region of eight individuals from each of the Ct-C and Ct-J clades. Amplification was successful, and the sequence length was 1610 bp (Additional file 3: Fig. S1). However, only three segregating sites were observed, and the 28S rDNA variants did not segregate with the observed clades.

### *Wolbachia *infection in *Cx. tritaeniorhynchus*

*Wolbachia* detection was initially performed by conducting PCR for the *wsp* gene. If *Wolbachia* was not detected, a second PCR targeting the 16S rDNA region was conducted. If amplification also failed in the second PCR, the result was considered to be negative, i.e. no *Wolbachia* DNA. The results from 12 regions of the ROK indicated that *Wolbachia* was detected in 32 (10.2%) of the 313 individuals of *Cx. tritaeniorhynchus* analyzed (Table 3). All 32 *Wolbachia*-positive individuals were from Ct-J, with none detected in the eight individuals from Ct-C.

**Table 3:** *Wolbachia* infection status of *Culex tritaeniorhynchus* in each population

Sites	Clade^a^	Sample size	Sample positive for *Wolbachia* (*n*)	Uninfected status(*n*)	Positivity rate (%)
Hoengseong	1 (Ct-C)	1	0	1	0
	2 (Ct-J)	6	0	6	0
Hwaseong	1 (Ct-C)	2	0	2	0
	2 (Ct-J)	16	0	16	0
Chungju	1 (Ct-C)	1	0	1	0
	2 (Ct-J)	30	3	27	10.0
Taean	2 (Ct-J)	6	0	6	0
Andong	1 (Ct-C)	1	0	1	0
	2 (Ct-J)	11	0	11	0
Wanju	1 (Ct-C)	1	0	1	0
	2 (Ct-J)	30	3	27	10.0
Daegu (2022)	2 (Ct-J)	29	8	21	27.6
Daegu (2023)	2 (Ct-J)	31	6	25	19.4
Gimhae	2 (Ct-J)	32	3	29	9.4
Sancheong	2 (Ct-J)	29	0	29	0
Gwangyang	2 (Ct-J)	30	0	30	0
Haenam	1 (Ct-C)	1	0	1	0
	2 (Ct-J)	44	9	35	20.5
Jeju	1 (Ct-C)	1	0	1	0
	2 (Ct-J)	11	0	11	0
Total	1 (Ct-C)	8	0	8	0
	2 (Ct-J)	305	32	273	10.5

^a^1 (Ct-C), Clade 1 *Cx. tritaeniorhynchus* continental type; 2 (Ct-J), clade 2 *Cx. tritaeniorhynchus* Japanese type

**Fig. 5:**
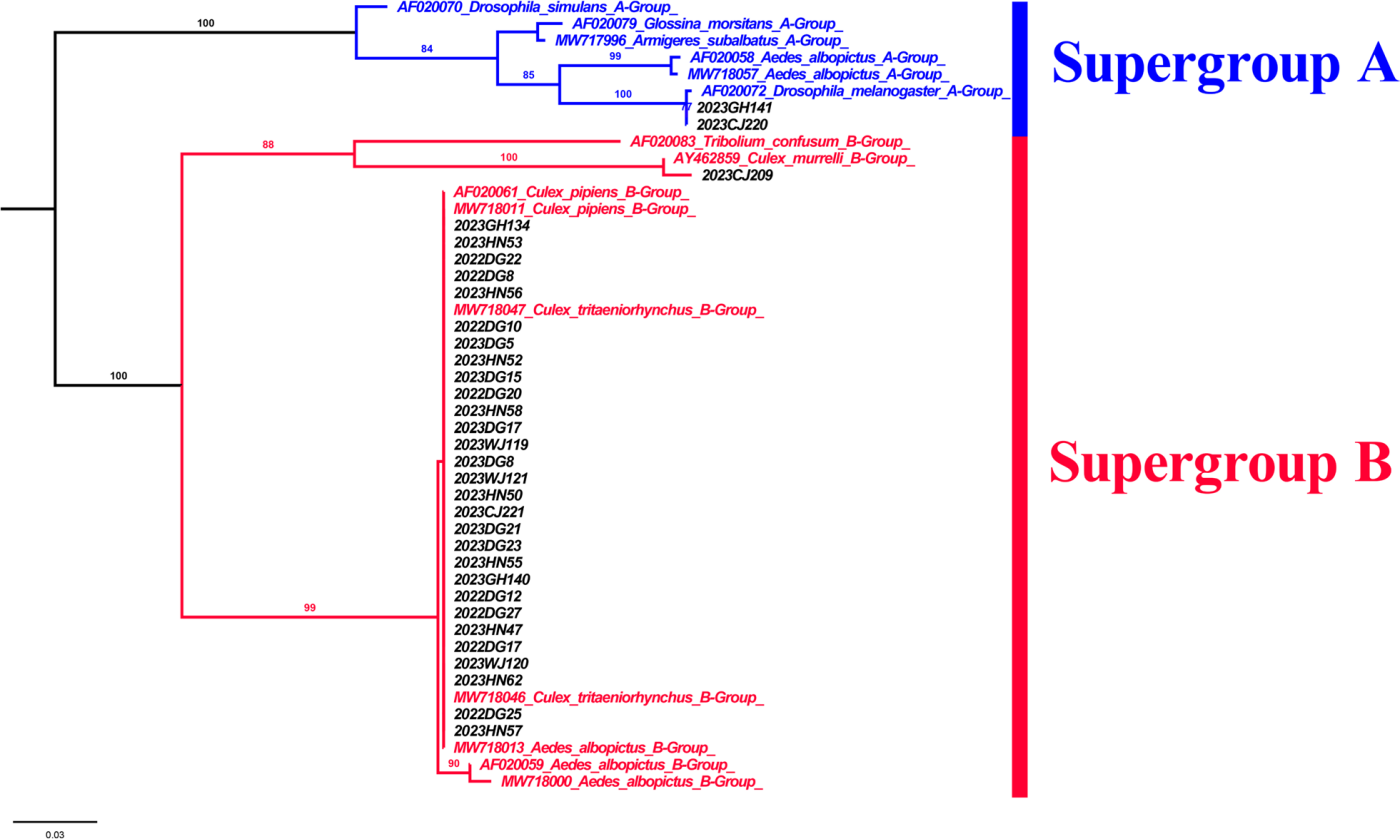
Mid-point rooted maximum likelihood phylogenetic tree constructed in this study using *Wolbachia* surface protein gene (*wsp*) sequences. Bootstrap analysis was performed with 1000 replications, and the bootstrap values are indicated on the branch. Individuals in supergroup A are shown in blue, and those in supergroup B are shown in red. Individuals labeled in black were obtained from this study. CJ Chungju; DG, Daegu; SC, Sancheong; WJ, Wanju

*Wolbachia* was identified in populations from five of the 12 regions. Phylogenetic analysis using ML based on the *wsp* sequence of *Wolbachia* from the infected mosquitoes confirmed the presence of supergroup A and supergroup B (Fig. 5). Among the 32 mosquitoes that tested positive for *Wolbachia*, two were infected with supergroup A and 30 were infected with Supergroup B. Supergroup A was found in Gimhae and Chungju, while supergroup B was identified in Gimhae, Chungju, Daegu (both in 2022 and 2023), Haenam and Wanju. Both supergroup A and supergroup B were present in Gimhae and Chungju. However, none of the 32 mosquitoes were found to be co-infected with both supergroup A and subgroup B.

### Possibility of invasion by *Cx. tritaeniorhynchus* from other countries

A comparison of the distribution of the Ct-C and Ct-J clades revealed that the ROK is predominantly inhabited mosquitoes of the Ct-J clade, with only a small number of Ct-C individuals found in certain regions.

To investigate the possibility of Ct-C being introduced to the ROK through air currents, a back trajectory analysis was conducted for the regions of Hwaseong, Haenam and Jeju, which are located near coastal areas where the Ct-C individuals were collected in the present study.

**Fig. 6:**
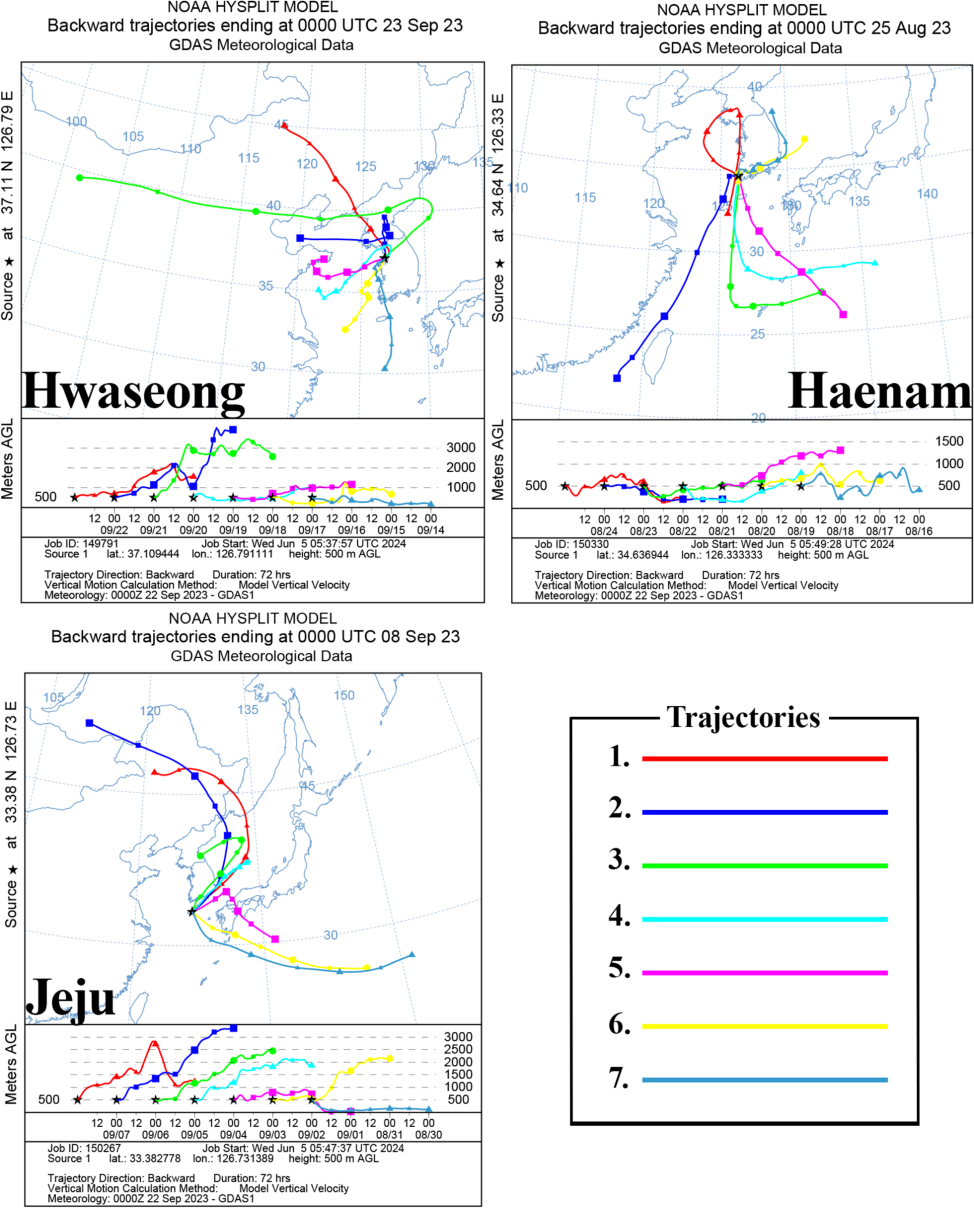
Results of back trajectory analysis for three regions (Hwaseong, Haenam, Jeju) in the Republic of Korea. Analyses were performed over a 7-day period from the day of collection, with 24-h intervals. The Global Data Assimilation System (GDAS) was used (https://www.arl.noaa.gov/hysplit/), with the arrival altitude set to 500 m. Each line (numbered 1–7) represents a distinct trajectory. The top portion of each image displays a horizontal path, while the bottom portion illustrates the elevation of the path

The analysis suggested the potential for the introduction of Ct-C individuals via air currents in all three regions at approximately the time of collection (Fig. 6). In Hwaseong, airflows generally originated from China, notably with a flow starting on 16–17 September 2023 (trajectory 5), reaching the ROK from China in approximately 2 days. In addition, the C-type individuals collected from Hwaseong and Wanju were confirmed to possess the same *COI* haplotype as the Ct-C specimens captured using the Johnson-Taylor suction trap by Arai et al. [14] (GenBank accession number: OK493320). The Johnson-Taylor suction trap is used as a tool for capturing long-distance migratory insects [14].

For Haenam, unlike Hwaseong, airflows originating from southern China and Taiwan (trajectory 2) were identified. In Jeju, an island region, the analysis suggested a possibility of introduction from Japan (trajectories 5, 6 and 7). However, the C-type collected in Jeju has been confirmed to share the same haplotype as the samples identified in Hangzhou (GenBank accession number: OK493345) and Jiangsu (GenBank accession number: OK465208) in China. These results suggest that it is possible that the introduction occurred from China to Japan and subsequently reintroduced to the ROK. C-type individuals identified in Hoengseong were found to share the same haplotype as those collected in Jiangsu, China (GenBank accession number: OK465245).

## Discussion

This study represents the first nationwide sampling carried out in the ROK to investigate the genetic structure of *Cx. tritaeniorhynchus*, the primary vector of JEV. The findings revealed the presence of two clades of *Cx. tritaeniorhynchus* in the ROK, with Ct-J being predominant. The AMOVA results indicated that genetic differentiation between regions was minimal in *Cx. tritaeniorhynchus*. This genetic feature has also been observed in *Cx. tritaeniorhynchus* populations in China [13], likely resulting from frequent genetic exchange between populations across different geographic regions, facilitated by *Cx. tritaeniorhynchus*’ exceptional flight capabilities [51]. In the ROK, this genetic structure is likely influenced not only by the mosquito’s flight ability but also by its capacity to overwinter. Average winter temperatures in the ROK are typically below 10 °C in most areas, which is unsuitable for *Cx. tritaeniorhynchus*, a mosquito species of tropical origin. This prediction is corroborated by studies on the overwintering behavior of *Cx. tritaeniorhynchus* in the ROK [52], which have found that the species overwinters only in a few coastal sites, with no individuals detected in inland areas [52], a pattern likely attributable to the ocean’s influence in moderating winter. Consequently, extensive gene exchange between populations is expected to occur as *Cx. tritaeniorhynchus* individuals that successfully overwinter in coastal areas migrate to repopulate inland and northern regions during spring and summer [53]. Future studies are necessary to investigate the implications of overwintering and the repopulation dynamics of *Cx. tritaeniorhynchus* during spring and summer in the ROK.

Based on an analysis of the *COI* region, Arai et al. [14] proposed that the *Cx. vishnui* subgroup may consist of four species: *Cx. vishnui*, *Cx. pseudovishnui*, *Cx. tritaeniorhynchus* (Ct-C) and *Cx. tritaeniorynchus* (Ct-J), rather than the previously recognized three species (*Cx. vishnui*, *Cx. pseudovishnui* and *Cx. tritaeniorhynchus*). Similar findings have been reported in China [13]. The potential existence of cryptic species within *Cx. tritaeniorhynchus* has also been suggested in India [54, 55]. However, comparisons of male genitalia, a key morphological characteristic for mosquito classification, revealed no significant differences between the Ct-C and Ct-J types [14]. In the present study, in addition to the analyzing the *COI* region, we also examined the rDNA region, specifically the 28S rDNA, and found no type-specific differences. In the near future, sequencing of 18S, another rDNA region not identified in the present study, and comparison to the mtDNA whole genome will be required. *COI* and rDNA regions are widely used genetic markers for species identification and differentiation [17, 21]. However, evidence suggests that these markers may not be reliable for certain mosquito groups, such as the *Culex pipiens* complex (subgroup) [27, 56, 57]. Therefore, species identification and population analyses in the *Cx. pipiens* complex have been conducted using various genetic markers other than *COI* and rDNA [58–61]. Accurate identification of disease vector species is the first step toward efficient vector disease control [62]. In the case of *Cx. tritaeniorhynchus*, comprehensive genome-wide data acquisition and analysis beyond the current *COI*- and rDNA-level analyses will be necessary to confirm the presence of cryptic species and ensure accurate classification. Additionally, it seems that a more comprehensive sampling effort should be conducted to include the various regions that were not examined in this research.

*Wolbachia* is a maternally inherited bacterium that significantly influences insect reproduction and disease transmission in vector species [63, 64]. The results of our study revealed that *Wolbachia* infections were present only in some individuals of the Ct-J clade. Both *Wolbachia* supergroup A and B were detected, with no evidence of clade-specific association. While certain regions exhibited high infection rates (Daegu, Haenam), the overall infection rate was relatively low (10.2%, 32/313), consistent with findings in China (17.1%, 14/82) [29]. The presence of *Wolbachia* in *Cx. tritaeniorhynchus* has also been documented in other Asian countries, including Thailand [65] and Singapore [66]. However, the reasons for the varying rates of *Wolbachia* infection in *Cx. tritaeniorhynchus* across different regions remain unclear. Furthermore, the effects of *Wolbachia* infection on phenotype and its role in disease transmission in *Cx. tritaeniorhynchus* also remain poorly understood.

The results of this study confirmed that only a small number of mosquitoes of the Ct-C clade are currently distributed in the ROK. Additionally, direct observation using microscopy, rather than sole reliance on PCR-based detection, may be necessary to confirm infection and investigate potential maternal transmission of *Wolbachia* [67, 68]. Currently, it appears that *Cx. tritaeniorhynchus* exhibits very low levels of *Wolbachia* infection in natural populations. Research into the relationship between *Cx. tritaeniorhynchus* and *Wolbachia*, including its potential antiviral effects, could inform future strategies for controlling *Cx. tritaeniorhynchus* using *Wolbachia* [69–71].

A recent study by Arai et al. [14] suggests that Ct-J is predominant in Japan, which is located in close proximity to the ROK (just across the Korea Strait), with Ct-C potentially being introduced into Japan via air currents. Similarly, the back trajectory analysis conducted in this study confirmed that in three of the regions analyzed (Hwaseong, Haenam and Jeju) there is a potential for Ct-C individuals to enter the ROK on air currents originating from different countries in the region, including China, Taiwan and Japan. Specifically, some of the airflow into Jeju was found to pass over the Kyushu region of Japan, where Ct-C has been identified [14]. Ct-C individuals were also detected inland in areas such as Hoengseong, Chungju, Andong and Wanju, rather than being found exclusively along the coast. For these inland occurrences, it is plausible that *Cx. tritaeniorhynchus* was initially introduced into coastal areas and subsequently spread inland [51]. This possibility can also be inferred from the fact that individuals of the Ct-C clade collected from Hwaseong and Wanju share the same haplotype. Alternatively, it is possible that a small number of Ct-C individuals have already established themselves in the ROK and coexist with Ct-J individuals. This finding underscores the need for ongoing monitoring of *Cx. tritaeniorhynchus* in both coastal areas where Ct-C individuals have been found and various inland locations. Therefore, developing molecular markers to differentiate Ct-C from Ct-J could be more cost-effective and allow for rapid identification compared to methods based solely on *COI* region sequencing. Currently, there is a lack of research elucidating the factors that contribute to the spread of Ct-C in the ROK and Japan, as opposed to the predominance of Ct-J on the mainland of these countries. Future research should focus on potential hybrids between the two clades of *Cx. tritaeniorhynchus*, the identification of reproductive barriers and investigations into behavioral and physiological aspects, such as vector competence, which are imperative for the effective prevention of JEV.

## Conclusions

Identification of cryptic species among disease vectors is crucial for controlling vector-borne diseases, particularly where individual species may need to be targeted. Hence, continuous surveillance and research on *Cx. tritaeniorhynchus*, the primary vector of JEV, is recommended.

## Supplementary Information

Additional file 1: Table S1. Collection sites information used in this study.

Additional file 2: Table S2. *COI* sequence information used in this study.

Additional file 3: Figure S3. 28S rDNA sequence alignments of *Cx. tritaeniorhynchus* obtained in this study.

## Data Availability

No datasets were generated or analysed during the current study.

## Abbreviations

AMOVA: Analysis of molecular variance
BLAST: Basic Local Alignment Search Tool
*COI*: Cytochrome* c* oxidase subunit I
Ct-C: *Culex tritaeniorhynchus* continental type
Ct-J: *Culex tritaeniorhynchus* Japanese type
JEV: Japanese encephalitis virus
ML: Maximum likelihood
mtDNA: Mitochondrial DNA
NOAA’s HYSPLIT: National Oceanic and Atmospheric Administration’s hybrid single-particle Lagrangian integrated trajectory
rDNA: Ribosomal DNA
ROK: Republic of Korea
*wsp*: *Wolbachia* surface protein
