# Supplementary material for: Genetic diversity and Wolbachia infection in the Japanese encephalitis virus vector Culex tritaeniorhynchus in the Republic of Korea
Source: Parasit Vectors. 2024 Dec 18;17:518. doi: 10.1186/s13071-024-06595-w (PMC11656722; doi:10.1186/s13071-024-06595-w)
Supplement: Supplementary file 1 — Additional file 1: Table S1. Collection sites information used in this study. [file 13071_2024_6595_MOESM1_ESM.docx]

**Table S1.** Collection sites information used in this study

| Collection sites | Coordinates |
| --- | --- |
| Hoengseong | 37°26'58"N, 128°03'08"E |
| Hwaseong | 37°06'34"N, 126°47'28"E |
| Chungju | 36°58'30"N, 127°46'29"E |
| Taean | 36°44'47"N, 126°13'46"E |
| Andong | 36°38'21"N, 128°43'51"E |
| Wanju | 36°00'04"N, 127°13'31"E |
| Daegu | 35°41'36"N, 128°24'02"E |
| Gimhae | 35°21'24"N, 128°51'03"E |
| Sancheong | 35°19'31"N, 127°59'54"E |
| Gwangyang | 35°00'12"N, 127°36'25"E |
| Haenam | 34°38'16"N, 126°19'48"E |
| Jeju | 33°20'32"N, 126°48'22"E |
