# Supplementary material for: Genetic diversity and Wolbachia infection in the Japanese encephalitis virus vector Culex tritaeniorhynchus in the Republic of Korea
Source: Parasit Vectors. 2024 Dec 18;17:518. doi: 10.1186/s13071-024-06595-w (PMC11656722; doi:10.1186/s13071-024-06595-w)
Supplement: Supplementary file 2 — Additional file 2: Table S2. COI sequence information used in this study. [file 13071_2024_6595_MOESM2_ESM.docx]

**Table S2.** *COI* sequence information used in this study

| Species | GenBank accession number |
| --- | --- |
| *Culex tritaeniorhynchus* (Ct-C) | OK493344, OK493345  OK493346, OK493347  OK493348, OK493349  OK493350, OK493351  OK493352, OK493353  OK493354, OK493355  OK493356, OK493357  OK493358, OK493359  OK493360, OK493342  OK493331, OK493328  OK493325, OK493320 |
| *Culex tritaeniohrynchus* (Ct-J) | OK493311, OK493312  OK493313, OK493314  OK493315, OK493316  OK493317, OK493318  OK493319, OK493321  OK493322, OK493323  OK493324, OK493326  OK493327, OK493329  OK493330, OK493332  OK493333, OK493334  OK493335, OK493336  OK493337, OK493338  OK493339, OK493340  OK493341, OK493343 |
| *Culex vishnui* | OK493366, OK493367  OK493368, OK493369  OK493370 |
| *Culex pseudovishnui* | OK493361, OK493362  OK493363 |
| *Culex orientalis* | LC646378 |
| *Culex bitaeniorhynchus* | OP927188 |
