# Supplementary material for: Genetic diversity and Wolbachia infection in the Japanese encephalitis virus vector Culex tritaeniorhynchus in the Republic of Korea
Source: Parasit Vectors. 2024 Dec 18;17:518. doi: 10.1186/s13071-024-06595-w (PMC11656722; doi:10.1186/s13071-024-06595-w)
Supplement: Supplementary file 3 — Additional file 3: Figure S3. 28S rDNA sequence alignments of Cx. tritaeniorhynchus obtained in this study. [file 13071_2024_6595_MOESM3_ESM.docx]

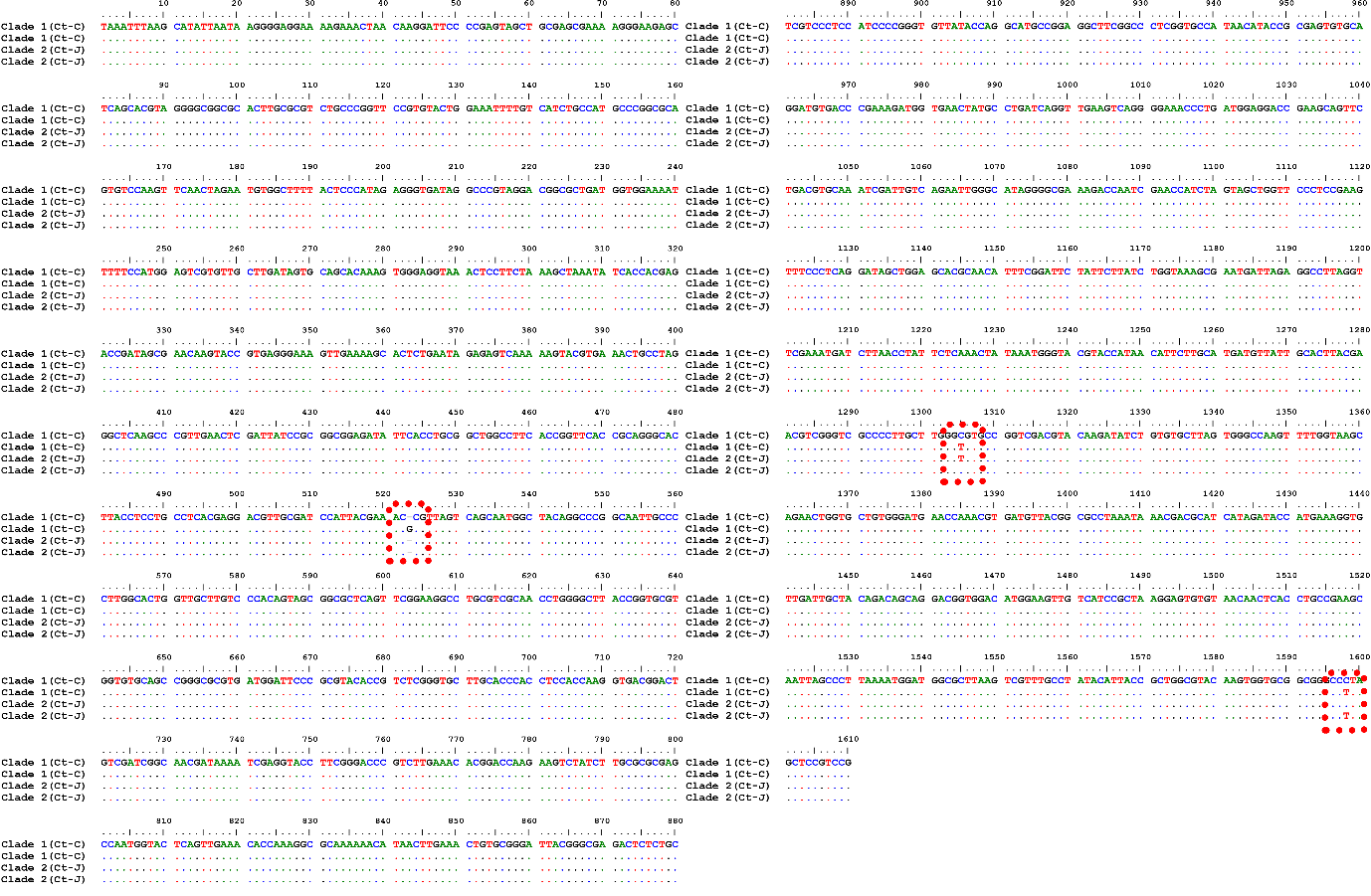


**Fig. S1.** 28S rDNA sequence alignments of *Cx. tritaeniorhynchus* obtained in this study. Variations are highlighted in red.
